# Irreversible electroporation of unresectable soft tissue tumors with vascular invasion: effective palliation

**DOI:** 10.1186/1471-2407-14-540

**Published:** 2014-07-26

**Authors:** Robert CG Martin, Prejesh Philips, Susan Ellis, David Hayes, Sandeep Bagla

**Affiliations:** Department of Surgery, Division of Surgical Oncology, University of Louisville, 315 E. Broadway - #312, 40202 Louisville, KY USA; Department of Interventional Radiology, Baptist Hospital, Little Rock, AK USA; Department of Interventional Radiology, INOVA, Alexandria, VA Egypt

**Keywords:** Irreversible electroporation, Locally advanced tumors, Vascular invasion, Liver tumors, Pancreatic tumors, Safety

## Abstract

**Background:**

Irreversible electroporation (IRE) has recently been added as an additional therapeutic ablative option in patients with locally advanced cancers (LAC) involving vital structures. IRE delivers localized electric current by peri-tumoral discrete probes to attain irreversible changes in cell membrane leading to cell death. The aim of this study was to evaluate the long-term effects of IRE in the treatment of locally advanced tumors.

**Methods:**

A prospective IRB approved evaluation of 107 consecutive patients from 7 institutions with tumors that had vascular invasion treated with IRE from 5/2010 to 1/2012. LAC was defined as primary tumor with <5 mm from major vascular structure based on pre-operative dynamic imaging or intra-operative criteria.

**Results:**

IRE as utilized in LAC in the liver (N = 42, 40%) and pancreas (N = 37, 35%), with a median number of lesions being 2 with a mean target size of 3 cm. IRE attributable morbidity rate was 13.3% (total 29.3%) with high-grade complications seen in 4.19% (total 12.6%). No significant vascular complications were seen, and of the high-grade complications, bleeding (2), biliary complications (3) and DVT/PE (3) were the most common. Complications were more likely with pancreatic lesions (p = 0.0001) and open surgery (p = 0.001). Calculated local recurrence free survival (LRFS) was 12.7 months with a median follow up of 26 months censured at last follow up. The tumor target size was inversely associated with recurrence free survival (b = 0.81, 95% CI: 1.6 to 4.7, p value = 0.02) but this did not have a significant overall survival impact.

**Conclusions:**

IRE represents a novel therapeutic option in patients with LAC involving vital structures that are not amenable to surgical resection. Acceptable to high local disease control and the long LRFS can be achieved with this therapy in combination with other multi-disciplinary therapies.

## Background

Electroporation is a phenomenon by which cell membrane integrity is compromised by inducing nanopores using trans-membrane electrical distortion. This was initially used to increase the permeability of the cells to therapeutic compounds and gene transfer in a reversible fashion [1]. Subsequently, it was used as an independent modality to achieve permanent cell destruction (irreversible electroporation) and demonstrated viability in cell, animal and later human models [2]. These studies confirmed that cell death occurred without breaching structural integrity and leaving vascular structures unharmed [3].

We and other authors have recently demonstrated the safety of the use of IRE around vascular and ductile structures on chronic large animal models [4–6]. Subsequent to those studies we have recently published organ specific safety and efficacy data with the use of IRE in liver and pancreas [7–9]. As with any novel technology in clinical practice, initial experience can be used to tailor subsequent indications, applications and strategies to limit the morbidity of the procedure. We present our multi-center experience with the single largest study of 107 patients who underwent IRE of soft tissue tumors with vascular invasion that were not amenable to surgical resection, thermal ablation and/or had failed radiation therapy.

## Methods

A prospective University of Louisville Institutional Review Board approved multi-institutional registry including University of Louisville Department of Surgery Division of Surgical Oncology, the Department of Interventional Radiology Baptist Hospital Little Rock AK, and The Department of Interventional Radiology INOVA Alexandria VA of consecutive patients undergoing IRE from 2010 through 2012 was reviewed. Additional sites that provide data were Cleveland Clinic Cleveland OH, Roper St Francis Hospital Charleston SC, Henry Ford Hospital Detroit MI, Stony Brook University Long Island NY. Ethical approval was obtained from all participating institutions. All patients provided written informed consented to include their data in this prospective data collection protocol. Inclusion criteria for this study included the presence of peri-vascular invasion of primary tumor (defined as tumor <5 mm from major vascular structure) based on pre-operative dynamic imaging or intra-operative criteria. The patient selection criteria, was left to the discretion of the treating physician and followed established guidelines that IRE should be utilized for locally advanced tumors that have failed initial standard therapy and demonstrate persistent local disease. General exclusion criteria, however, were any contraindications for general anesthesia, extensive extra-organ of ablation disease, or multifocal hepatic disease not amenable to complete ablation.

IRE was performed using the Angiodynamics Nanoknife system (Angiodynamics, Latham, NY). The Nanoknife system consists of a computer controlled pulse generator that delivers 3000-volt pulses to the IRE probes. The pulse voltages and duration are based on preclinical studies [4, 10] as well as clinical studies [10, 11]. The procedure itself was similar to that described in our previous experience. Typically a minimum of 90 pulses is delivered which last from 20 to 100 microseconds each. The most common pulse duration is 90 microseconds, although shorter durations (70 or 80 microseconds) may be utilized in cases where high electrical resistance is encountered. Treatment planning is based on preoperative imaging with CT scanning in which the tumor dimensions and morphology are measured. Tumor dimensions are then measures and the number and spacing of probes needed to create the desired ablation zone based on the instruction for use are utilized. The needles are multiple monopolar 19-gauge radio-opaque probes, spaced 1.5 to 2.2 cm apart, were used depending on the electroporation zone to be achieved.

Access for the IRE procedure itself was percutaneous, laparoscopic or open depending on the preference of surgeon or interventional radiologist. CT guidance was used for all percutaneous cases. General anesthesia with deep neuromuscular blockade was used in all cases to achieve paralysis to 0 twitches out of a train of 4. This level of paralysis is needed to prevent patient movement when the high voltage pulses are delivered. When multiple probe arrays are utilized, a mechanical guide (spacer) is employed to maintain proper spacing and alignment. The probes are placed in a manner as to bracket the tumor, rather than violate the tumor itself. The probes must also be completely encased in tissue to prevent arcing.

Ablation technical success was defined as the ability to successful deliver all planned pulses (at least 90) in accordance with size and dimension of the lesion, as well as on at least 12 week axial scanning to demonstrate a complete ablation without evidence of enhancement. The definition of proximity to major vascular/biliary structures or adjacent organs was defined as <5 mm in distance.

Adverse events were recorded as per the established Common Terminology Criteria for Adverse Events (CTCAE), version 3.0. All complications were recorded prospectively at all institutions. Follow up imaging was performed at the time of discharge or with 2 weeks of IRE therapy for safety evaluation and then at three-month intervals. Early scans were obtained to look for complications such as portal vein thrombosis. Imaging was ordered by the treating physician and/or the multidisciplinary team caring for the patients. Post-ablation recurrence was defined as persistent viable tumor as defined by dynamic imaging in comparison to pre-IRE scan or tissue diagnosis. Ablation success was defined as the ability to deliver the planned therapy in the operative room and at 3 months to have no evidence of residual tumor as described above. The method of evaluating local recurrence is the combination use of both cross-sectional imaging, either a CT scan or MRI, with or without PET scanning based on 1) the ability to obtain a preoperative PET scan and 2) that the primary lesion in question had PET activity. In cases where preoperative PET scan was obtained and the lesion was PET avid, persistent or recurrent PET avidity was evidence for tumor recurrence. Specific cutoffs for SUV to determine recurrence were not utilized. The use of CT versus MRI imaging for follow-up was left to the discretion of the treating physician. Dedicated body-imaging radiologists, who were not blinded to treatment, made radiologic interpretation of recurrence. As noted above, general radiologic criteria for recurrence are new or persistent enhancement on multi-phase imaging such as defined by the RECIST criteria [12]. In cases where imaging was equivocal, biopsies were obtained at the discretion of the treating physician.

Patient demographics, tumor characteristics, in hospital outcomes, and local recurrence free survival were examined. Continuous variables were summarized by median and interquartile range (IQR) and compared using the Wilcoxon-Mann–Whitney test while categorical variables were summarized as count (percentage) and analyzed using the chi-squared or Fisher’s exact test, where appropriate. Local recurrence free survival (LRFS) was determined from the time of ablation to radiographic recurrence of the treated lesion. Patients without evidence of recurrence were censored at the time of last follow-up. Survival estimates were determined according to the method of Kaplan and Meier, with survival curves compared by the log rank test. The relation of target lesion size to LRFS was determined according to Cox proportional hazards regression. To determine whether there was an appropriate cutoff in tumor size related to increased risk of LRFS, plots of martingale residuals versus tumor size were examined as described [13]. All statistical analyses were performed using SPSS version 20.0, with p < 0.05 considered significant.

## Results

A total of 107 consecutive patients underwent 117 IRE procedures for tumors with vascular invasion from May 2010 to January 2012 from 7 centers. The median age for this cohort was 62 years (mean 62.14, SD 12.2) with a slight male predominance (50.4%). Diabetes was noted in 20 (18.7%), and a significant history of cardiac disease or pulmonary disease was seen in 8 (7.4%) patients each. Tobacco use was prevalent in 35 (32.7%) while alcohol abuse was found in 7 (6.6%) patients. Prior history of hepatitis and pancreatitis was seen in 5 (4.7%) each. In 67 (57.2%) patients a history of prior abdominal surgery was recorded. Median BMI was 26.9 and Karnofsky score was 90%.

**Figure 1:**
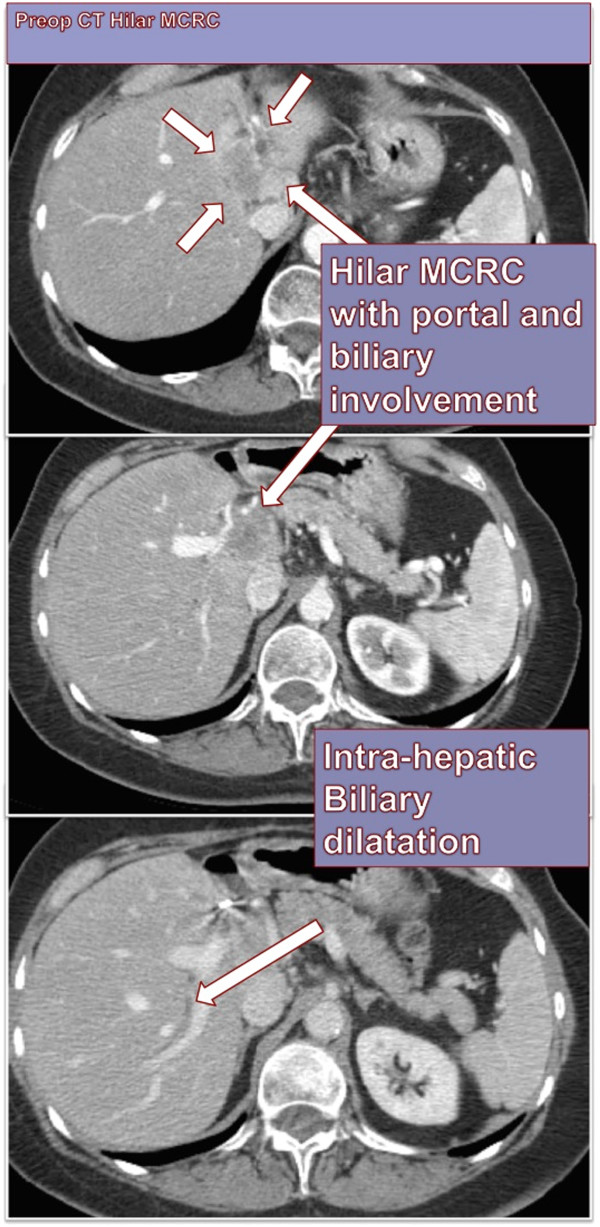
**A representative target lesion treated in this series with clear vascular invasion of the liver hilum in a patient with metastatic colorectal cancer.**

The access for IRE itself was either through a laparotomy in a majority of the cases (81–69.2%), or percutaneous CT guided in 32 (27%) and in 3 cases laparoscopy was used for access. Majority of these were pancreatic cancers (n = 84, 72%, 75 were performed through open incision) and liver lesions constituted (n = 17, 14.5%) with the rest being lung, kidney, mediastinal, pelvic and prostate. Vascular structures proximity was confirmed with pre-operative imaging (Figure 1). Liver lesions were mostly colorectal hepatic metastasis (n = 11, 64%) and pancreatic lesions were adenocarcinoma (n = 76, 90.4%) of the head and body. Among the 81 cases performed by laparotomy there were 56 (69.1%) associated major alimentary or hepatobiliary procedures. Pancreatic resections (total, Whipple, distal pancreatectomy) comprised 23 (41%) of the major procedures and hepatectomy was done in 5 (9%). Enteric bypass and bilio-enteric anastomosis were performed in 42 (77%) and splenectomy in 7 (13.4%). Vascular reconstruction was done in 14 cases (12%), with IRE performed prior to resection in order to accentuate the surgical resection margin. The decision to perform resection with IRE was based intra-operatively if after surgical exploration and dissection that an R1 resection would occur. IRE with resection WAS NOT neither performed nor recommended if an R2 resection would have occurred. Other minor procedures included cholecystectomy (n = 17), feeding access (n = 32) and celiac axis block (n = 10).

In a majority of the pancreatic lesions, vascular invasion was to the portal vein (n = 70), to the superior mesenteric artery or vein (n = 19), and the celiac axis (n = 6). For the hepatic lesions, site of vascular invasion was the portal and/or the hepatic veins.

A history of prior chemotherapy was seen in 82 (76.7%) of patients, while radiation history was noted in 67 (63%) patients. Other locally ablative procedures (RFA, ethanol, microwave) had been previously tried in 11 (10.2%). Hepatic arterial therapy such as TACE, Yt^90^ and bland embolization was previously tried in 10 (9.3%).

Median size of the lesion in X, Y and Z-axis was 3 × 2.5 × 2.75 cm each with a mean of 3.14 × 3 × 2.8 cm. Mean and median total target size was 3.5 and 3.66 cm. Median number of lesions ablated per patient was 2. Some patients (10, 9.3%) had lesions that were numerous and could not be counted accurately but were within a localized ablation/ resection zone.

Median procedure time was 170 minutes (Mean: 174 + -97, IQR 109) while the actual probe placement time was 10 mins (mean 15, IQR 5–20) and IRE delivery time per ablation was 28 (Mean 43.7, IQR 14–75) minutes. Concurrent major abdominal procedures in 54 (50.4%) patients were associated with an increased operative time (195 vs. 114 minutes, p < 0.0001) but similar actual IRE delivery time (30 vs. 28 mins). Number of pulses delivered was 90 and median number of probes used per ablation was 3 with a mean of 3.35 with lesion overlap seen in 51 (47.6%) cases. In 29 (31%) treatments high current conditions were noted. In 7 the exact reason for this was unknown but treatment was completed in a majority of cases. Of the rest 22 the reason was either retreatment area (which imparts high current situations) or dense tissue with a minority due to sub-optimal probe spacing.

Peri-procedural electroporation complications (defined as within 90 days of IRE) were graded per CTCAE version 3.0. There were a total 43/107 (40%) patients with 84 complications. The median complication grade was 2 (Table 1). There were no reports of procedure induced dysrhythmias or major intraoperative bleeding in this cohort. Infectious complications included wound infection (5/107, 4.7%), UTI (3/107, 2.8%), intra-abdominal abscess (2/107, 1.9%) and pneumonia (1/107, 0.9%). Peri-operative nausea and vomiting was seen in 7/107 (6.5%) and 3/107 (2.8%) patient’s experienced prolonged ileus. There were 3/107 (2.8%) patients with vascular complications namely, 1 case of portal vein thrombus, superior mesenteric artery vein thrombus and one of hepatic artery thrombus, each. Two of these patients were on prior anti-coagulation, one from extremity venous thrombosis and the other from a old history (5 months prior to IRE) of a pulmonary embolus. Significant biliary complications included 2/107 (1.9%) cases of bile leak and 3/107 (2.8%) biliary strictures. There were also 2/84 (2.4%) associated pancreatic leaks and 3/84 (3.6%) cases of duodenal fistula or leak. Transient liver failure was seen in 2/17 (11.9%) and temporary renal failure was seen in 3/107 (2.8%).

**Table 1:** **Factors affecting complications and high-grade complications after IRE (vascular invasion)**

	Complications (# Pts with complications)	High-Grade complications	Univariate analysis (p value)*	Multivariate		
				Hazard ratio	95% Confidence interval	p value
**#Pts with PMH cardiac (n = 16pts)**	6 (38%)	2 (13%)	0.4/0.7			
**#Pts with PMH Diabetes (26pts)**	8 (31%)	2	0.009/0.05^	4.0	1.4-11	0.007^
**#Pts with Tobacco use (40 pts)**	12 (30%)	4	0.2/0.5			
**#Pts with Pancreas IRE(59 pts)**	30 (51%)	12	0.01/0.4^	3.2	1.3-7	0.01^
**#Pts with Open IRE (60 pts)**	34 (57%)	13	0.00/0.00^	5.8	1.4-11	0.02^
**#Pts with Major Abdominal Procedure (56 pts)**	27 (48%)	15	0.03/0.2^	0.8	0.3-1.8	0.5
**#Pts with Radiation (67 pts)**	21 (31%)	16	0.02/0.09^	3.6	1.3-9	0.01^
**#Pts with prior Intra-arterial Rx# (21 pts)**	2 (10%)	1	0.3/0.4			
**#Pt with prior ChemoRx (106 pts)**	30 (28%)	11	0.8/0.4			
**Size of lesion > 3 cm**	26 (36.1%)	9 (12.5%)	0.01/0.4^	1.3	0.6-1.9	0.8

# Intra-arterial emboic therapy (SIRS, TACE, Bland), ^Higher risk of complications.

*Univariate analysis: 1^st^ value is p value for complications, 2^nd^ value is p for high-grade complications.

High-grade complications were seen in 21 patients with a high-grade complication rate of 17.9%. Complications were also divided into complications that were related to the IRE or those related to associated procedures. Related (attributable) complications were seen in 19 (16.2%) patients and high-grade attributable complications in 6 (5.1%).

Factors associated with complications were analyzed. Diabetes was associated with an increased overall complication rate (p = 0.009) and high-grade complication rate (p = 0.05). Other medical co-morbidities including prior cardio-pulmonary disease, tobacco and alcohol use or prior abdominal surgery did not statistically affect the complication rates or high-grade complications. There was a higher incidence of complications with pancreatic lesions (p value = 0.001). Laparotomy for access (n = 81, p < 0.0001) and concurrent major abdominal procedures (p = 0.02) significantly increased the complication rates. A prior history of radiation was predictive of complications (p value = 0.01) while percutaneous procedures (p < 0.0001) and colorectal-hepatic metastases (CRHM, p = 0.01) were associated with significantly lower rates. Prior or recent chemotherapy, intra-arterial therapy and previous abdominal surgery did not impact the complication rate. Complication rates were lower in patients who underwent prior ablation or resections (p = 0.01) but this was primarily due to the fact that these patients had liver lesions.

A total of 2 (0.9% one related -0.4%) peri-operative (within 90 days from IRE) deaths were seen in this study group. One was felt to be possible related to IRE from VTE, the other was not related to IRE and was from urinary sepsis that was treated at an outside facility and was decide to initiate hospice care only. Analysis was performed and a diagnosis of pancreatic cancer and size of the lesion (p = 0.01) was noted to be a significant association whereas vascular invasion, size of the lesion and prior chemotherapy was not noted to be statistically significant factors.

After a median follow up of 29 months 39 (23%) had recurrence of disease – local or distant. Mean time to recurrence was 9.9 months in those patients that had recurrence (local and remote), for liver median time was 12 months (range 4 to 18 months) and for pancreas median was 16 months (range 3 to 36 months). The recurrences were diagnosed with CT scan (n = 25), MRI (n = 7) and PET CT (n = 7), for patients who had a PET positive lesion pre-procedure, and 3 patients had biopsy confirmation. Seven (5.9%) patients had evidence of recurrence at their 3 month follow up imaging and were called persistent disease and underwent re-ablation.

**Figure 2:**
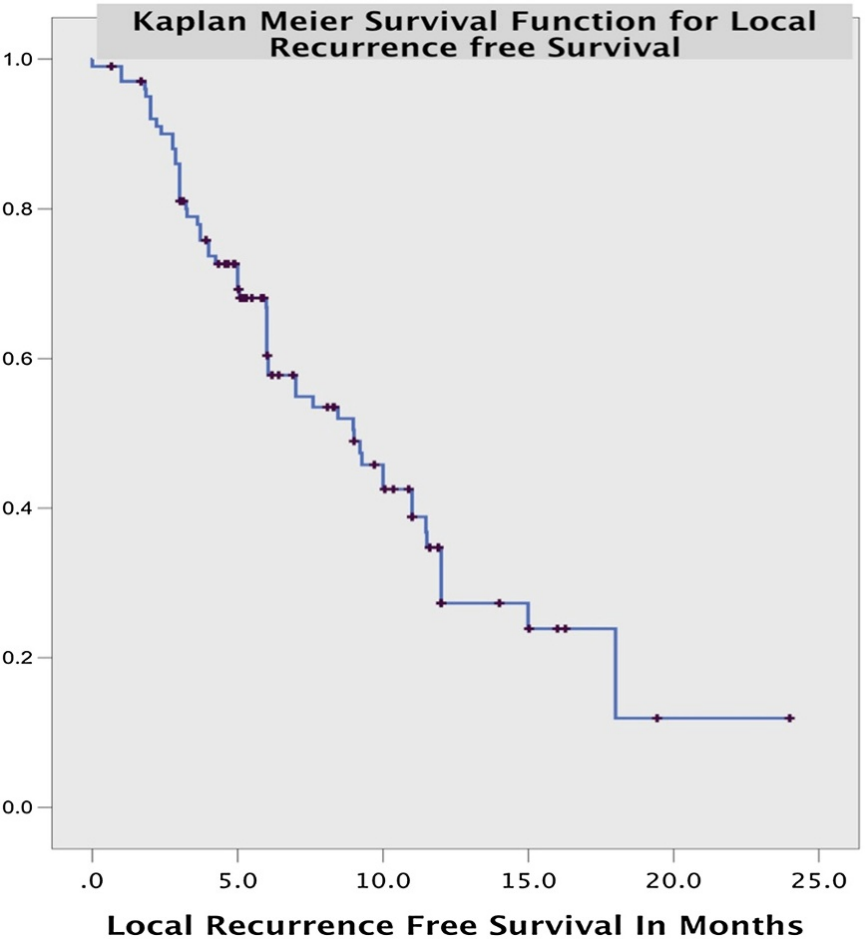
**Overall local recurrence free survival for all target lesions treated with IRE.**

The calculated LRFS (local recurrence free survival) was 12.7 months for the entire cohort (Figure 2). Analysis showed that presence of nodal disease, incomplete first treatment, and adverse events at 1st treatment decreased the recurrence free survival significantly. The tumor target size was inversely associated with recurrence free survival (b = 0.81, 95% CI: 1.6 to 4.7, p value = 0.02) but this did not have a significant overall survival impact. Factors such as organ of lesion, medical co-morbidities, access for IRE, vascular invasion and prior adjuvant therapy had no impact on RFS (Table 2).

**Table 2:** **Factors affecting local recurrence free survival after IRE**

	P value	Hazard ratio	95% CI	
			Lower	Upper
**Open procedure**	0.12	0.34	0.017	0.77
**Incomplete ablation**	0.18	0.386	0.1-1.5	1.53
MCRC*	0.05*	0.3	0.01	0.96
**Pancreatic Adenocarcinoma**	0.7	1.251	.322	4.85
**Total Number of Lesions**	.510	.794	.401	1.57
**Largest Dimension >3 cm^**	0.02^	2.02	1.1	3.6
**Chemotherapy^**	0.05^	2.82	1.006	8.02
**Radiation**	0.1	0.5	0.2-1.2	1.2

*Lower risk of recurrence, ^Higher risk of recurrence.

Regarding mortality data and overall survival, 54 patients were deceased at the time of this analysis with a median follow-up of 18 months. In all patients at follow up, there has not been any evidence of vascular stricture or narrowing of the vital structure in question. We have seen 4 patients who developed portal/superior mesenteric thrombosis during follow up (median time to thrombosis was 6 months, range 3–10 months) with currently no evidence of recurrent disease.

Analysis showed that factors associated with a worse prognosis with respect to overall survival and mortality included a diagnosis of pancreatic lesion (p = 0.00), and the presence of serious adverse events in the first treatment (p = 0.00). Age had no impact on survival (p value = 0.521), nor did a history of prior cardiac/pulmonary disease (p value = 0.2), diabetes (p value = 0.9), vascular disease (p value = 0.8), hypertension (p value = 0.2) or a history of abdominal surgery (p value = 0.9).

The presence of a recurrence did not affect overall survival in our study (p value = 0.26). Other factors that did not affect overall survival were abnormal parenchyma (p value = 0.9), nodal disease (p value = 0.8), peritoneal disease (p value = 0.9), prior chemotherapy (p value = 0.8), recent chemotherapy (p value = 0.7) and incomplete first treatment (p value = 0.68).

A total of 12 patients had incompletely ablated tumors. These observations were made of some of the incompletely ablation patients:

-  A presacral mass with persistent activity in the neural foramen
-  Inferior margin of lung mass
-  Disease adjacent to RLL bronchus
-  2 pancreatic body
-  2 had good result following repeat IRE
-  2 ablations could not be completed due to pacer problems or mechanical difficulties.

Size of the lesion, number of lesions and histology did not have a significant impact on presence of incomplete ablation.

## Discussion

Irreversible electroporation is a relatively new and evolving technique in soft tissue tumor ablations and palliation [14]. Its advantages compared to RFA, microwave, and cryotherapy are its non-thermal delivery mechanism. When IRE is delivered appropriately it only affects the target tissue and spares the surrounding structures. Proteins, the extracellular matrix, and critical structures such as blood vessels and nerves are all unaffected and left healthy by this treatment [2]. IRE expands the scope of palliative and/or definitive treatment of lesions near major vascular/biliary/urinary structures that in the past could only be treated with some forms of external beam radiation therapy. The disadvantage is the need for general anesthesia (deep paralysis) for its safe and effective delivery [8].

This study is the single largest prospective evaluation of IRE therapy in various organ sites and across access techniques. Our data demonstrates acceptable safety and optimal local disease control when used by the appropriately trained physician.

The safety of IRE in this study group was evident with this older population of a median age 62 years old, which is comparable to other tissue ablation experiences [15, 16]. Cardiopulmonary disease was seen in 20.7% and a history of tobacco use in 27%, which is higher than with other reports of early RFA studies [17]. Even in the population of patients with cardiac disease there was not a single episode of cardiac toxicity (i.e. ventricular arrhythmia, tachyarrythmias, or atrial fibrillation that prevented effective energy delivery). In our study the incidence of cirrhosis was lower than previously reported but this is a reflection of the distribution of lesions among other organs and a higher incidence of metastatic lesions that were ablated among liver lesions [15]. Majority of the cases involved liver and pancreatic lesions (75), with the access for IRE itself through a laparotomy (81 = 69.2%), percutaneous CT, 32 (27%), or laparoscopy (N = 3). The relatively large proportion of procedures done is more a reflection of individual and institutional bias with some centers having a significantly higher proportion of open procedures. A significant number or patients (22%) had other associated procedures, which also explains the high number of patients who underwent open procedures. This is in contradistinction to the early learning curve analyses of RFA, which evaluated primarily percutaneous RFAs without any other associated procedures. We chose to present our consolidated data to reflect the individual preferences of the operators as well as to evaluate this new procedure in its varied access and organ-specific approaches.

As far as the distribution of liver lesions, there were more metastases ablated than primary liver tumors, which is in contrast with comparative non-western studies [15] but similar to recent western literature for RFAs [18]. The mean number of lesions was also similar to comparative studies. A significantly higher number of tumors were noted to have a vascular invasion, which is defined as being less than 5 mm of major vascular structures. This was much higher than most studies of similar ablative techniques and is reflective of more advanced disease and the advantage of IRE’s non-thermal action that allows it to be used near vascular structures without significant complications. Lesions with significant vascular involvement or involvement of biliary, collecting system, bronchial tree and neural structures have long been noted to be significant contraindication for traditional thermal induced ablation techniques. IRE offers a suitable alternative and in this study we found a large number such anatomically hostile lesions. In spite of this with respect to vascular complications, only one case of portal vein thrombosis worsening in a patient with preexisting portal vein thrombus was noted with a vascular complication rate of 1.3% (1/77 cases) in patients with vascular invasion and 0.6% in this cohort. This rate is significantly lower than similar studies in RFA despite the low rate of vascular proximity in those lesions, demonstrating the safety of IRE in this situation [15, 18].

A total of 452 lesions were ablated with median number of ablations per treatment being 2 with mean dimensions of 2.49 × 2.24 × 2 cm. The procedure time at 152 minutes was significantly longer than most in relatable thermo-ablative studies and similar to IRE studies, despite the high number of associated procedures [17]. Delivery of 90 pulsed treatments with an average of 2 mins per treatment lends to a significantly longer treatment time than RFA and microwave, and is one of the disadvantages of IRE.

Incomplete ablation was noted in 12 (4.7%) lesion ablations, 5 of which were subsequently ablated adequately. In the other cases it was felt by the operator that lesion was in an anatomically unfavorable position, leading to poor lead placement and incomplete delivery. This is a high percentage of lesions that were either incompletely ablated or found unsuitable. As noted in our previous experience, pre-operative dynamic imaging which is used to plan these treatments, sometimes underestimates the degree of involvement with surrounding structures or the size, especially for pancreatic and retroperitoneal structures. A majority of our patients had a post-procedure imaging and one at 3 months to evaluate the response to treatment and 10.1% of patients had evidence of persistent tumor on repeat imaging. 11 of who underwent re-ablation successfully. This is a rate that is similar to initial RFA learning curve experiences [18], but most studies did not report this rate.

The results that we have presented compare favorable to High Intensity Focus Ultrasound (HIFU), which continues to report an ablation success of 91%, 79%, and 50% from the most current series reported in large number of patients [19–21]. That inferior ablation success reported above, coupled with ablation recurrences of 35%, 21%, and 28%, make IRE a potential more superior local palliative option with better ablation success and long term disease control.

The complication rate in this cohort was 29.3%, which is significantly higher than similar studies (reported complication rates of (6-16%) in RFA and microwave. Complications were also graded as related to the procedure, unrelated, possibly related, and related to associated procedures. For purposes of analysis IRE related complications were all complications that were related, possibly related and those without any good causative associations. Analysis of IRE related complications took this rate down to 13.3%, which is more congruent with similar precedents. On comparing only percutaneous IRE and their complication rates, the complication rate was 6.8%, which is similar to precedents. High-grade complications were noted in 16 (10.6%) with 6 (4%) IRE related complications attributable to IRE. No specific gradation of complications and auditing of re-interventions were seen in similar studies, such that a comparison could not be made. There were no cases of cardiac arrhythmias in this study, which is lower than previous literature [17].

Local recurrence was seen in 10.7% of patients ablated and is comparable to similar studies. A LRFS of 9.7 months is hard to interpret with this anatomically and biologically diverse lesion, but in view of the nature of uniformly advanced disease and it is congruent with other studies. These lesions were larger lesions with greater numbers of anatomically hostile features including vascular invasion and we think that this is an acceptable recurrence rate given the mean follow up period. Larger lesions, advanced local disease (nodal and peritoneal disease) led to greater recurrences. As expected an incomplete first treatment (even if subsequently addressed) as well as adverse events at ablation led to shortening of LRFS.

## Conclusions

IRE is a new non-thermal based electroporation technique of tissue ablation, which acts by changing the membrane properties allowing cell death. Accurate mapping and image-based guidance can lead to precisely targeted tissue destruction. Since it is not thermal based, it avoids the "sump" limitations and can be used for lesions abutting thermo-sensitive or thermal-limiting structures such as vascular, biliary, urinary and nervous structures. A multi-center analysis of these patients, with a variety of lesions and access techniques demonstrated that IRE could be successfully performed in a majority of the cases without major adverse events. As expected tumor biology, with respect to the organ of origin of the lesion was a significant factor with respect to mortality and overall survival. Institutional and individual preferences colored the mode of access and other associated procedures that were performed simultaneously. With time, more complex treatments of larger lesions and lesions with greater vascular involvement was performed without a significant increase in adverse effects or impact on LRFS. The evolution of this procedure over time in this initial experience demonstrates the safety profile of IRE and the relative speed of graduation to more complex lesions in a relatively short span of time.
